# Impact of various microplastics on the morphological characteristics and nutrition of the young generation of beech (*Fagus sylvatica* L.)

**DOI:** 10.1038/s41598-024-70046-w

**Published:** 2024-08-20

**Authors:** Jarosław Lasota, Ewa Błońska, Marta Kempf, Piotr Kempf, Sylwester Tabor

**Affiliations:** 1 Department of Ecology and Silviculture, Faculty of Forestry, University of Agriculture in Krakow, 29 Listopada 46 Str., 31-425 Kraków, Poland; 2 Krakow Regional Directorate of State Forests, Juliusza Słowackiego 17a, 31-159 Kraków, Poland; 3 Department of Machinery Exploitation, Ergonomics and Production Processes, University of Agriculture in Krakow, ul. Balicka 116B, 31-149 Kraków, Poland

**Keywords:** Beech seedlings, Forest management, Plastic pollution, Stoichiometry, Environmental health, Plant development, Plant stress responses

## Abstract

Microplastics have the capacity to accumulate in soil due to their high resistance to degradation, consequently altering soil properties and influencing plant growth. This study focused on assessing the impact of various types and doses of microplastics on beech seedling growth. In our experiment, we used polypropylene and styrene granules with diameter of 4.0 mm in quantities of 2.5% and 7%. The hypothesis was that microplastics significantly affect seedlings' nutritional status and growth characteristics. The research analysed seedlings' nutrition, root morphological features, above-ground growth, and enzymatic activity in the substrate. Results confirmed the importance of microplastics in shaping the nutritional status of young beech trees. Microplastic type significantly impacted N/P and Ca/Mg stoichiometry, while microplastic quantity influenced Ca/Al and Ca^+^K^+^Mg/Al stoichiometry. Notably, only in the case of root diameter were significantly thicker roots noted in the control variant, whereas microplastics played a role in shaping the leaves' characteristics of the species studied. The leaf area was significantly larger in the control variant compared to the variant with polypropylene in the amount of 2.5% and styrene in the amount of 7%. Additionally, the study indicates a significant impact of microplastics on enzyme activity. In the case of CB and SP, the activity was twice as high in the control variant compared to the variants with microplastics. In the case of BG, the activity in the control variant was higher in relation to the variants used in the experiment. Research on the impact of microplastics on the growth of beech seedlings is crucial for enhancing our understanding of the effects of environmental pollution on forest ecosystems. Such studies are integral in shaping forestry management practices and fostering a broader public understanding of the ecological implications of plastic pollution.

## Introduction

Microplastics, tiny fragments of plastic less than 5 mm in length, pose a significant environmental challenge, especially in soil ecosystems^1–3^. These slight particles originate from various sources, including the breakdown of larger plastic debris, microbeads in personal care products, synthetic fibres from clothing, and abrasion of tires and industrial materials^4^. In soil environments, microplastics are particularly concerning for several reasons. Microplastics can accumulate in the soil, as they are highly resistant to degradation. This accumulation can alter the physical properties of the soil, affecting its structure, porosity, and water retention capabilities^5–8^. This alteration can impact root growth and soil aeration, which is crucial for healthy plant development. Microplastics in soil can harm living organisms, such as earthworms, insects, and microorganisms^9,10^. These organisms play crucial roles in soil health and fertility, and their impairment can disrupt the balance of the ecosystem^11,12^. Microplastics can adsorb and concentrate environmental pollutants like pesticides and heavy metals^13^. These chemicals may then be released into the soil, posing further risks to soil health and potentially entering the food chain^14^. Microplastics in soil can affect plant productivity. Their presence can interfere with plant growth and health and plants risk-taking up these plastics^15,16^. Removing microplastics from soil is challenging due to their small size, widespread distribution, and the complexity of soil matrices^17^. Some studies have found that microplastics can affect root architecture^18^. This can lead to reduced plant growth and lower yields, as roots are essential for water and nutrient uptake. Microplastics in soil may interfere with the absorption of nutrients by plants^16^. The particles can bind with nutrients, making them less available to plants, resulting in nutrient deficiencies and affecting plant health and growth. Microplastics can lead to disturbances in the balance of nutrients in the soil, limiting the diversity and activity of microbiological populations^19^. Microplastics can impact the microbial communities in the soil, which play a vital role in nutrient cycling and plant growth^20^. Microbial activity and diversity changes can affect the availability of essential nutrients to plants^21^. The effect of microplastics varies among different plant species. Some plants may be more susceptible to the negative impacts of microplastics, while others may show little or no effect^22–24^. The findings of Yang et al.^25^ demonstrated that microplastics inhibit plant root development and the diversity and function of rhizosphere bacteria.

So far, little research has been conducted on the impact of microplastics on trees used in forestry. Our understanding of how soil microplastic contamination impacts the growth of young trees is limited. Our research aimed to determine the impact of different types and different amounts of microplastics on beech seedlings' nutrition and growth characteristics. Beech is an important species in temperate forest stands that is expanding its range as the climate changes^26^. We assumed that the type and amount of microplastics affect the nutritional status of seedlings and, to a lesser extent, the morphological features of the root systems of beech seedlings. Additionally, we assumed that the presence of microplastics influenced the enzymatic activity of the substrate in which the beech seedlings grew. Investigating the effects of microplastics on beech seedlings can provide crucial insights into how these pollutants influence forest ecosystems. Beech trees are significant components of many temperate forests, and understanding their interaction with microplastics can reveal broader ecological implications.

## Materials and methods

### Study sites and experiment design

The research was conducted at a forest nursery in the Wisła Forest District in southern Poland (49° 32′ 04″ N; 18° 55′ 59″ E). The study focused on beech (*Fagus sylvatica* L.), which was sown in five different types of substrates. The first variant was control (C)—a standard peat substrate used in nurseries. The physicochemical properties of the experimental substrate used were analysed (Table 1). The second and third variants (PP2.5 and PP7) were substrates with the addition of polypropylene in the amounts of 2.5% and 7% in relation to substrate volume, respectively. The fourth and fifth variants (ST2.5 and ST7) included a substrate with 2.5% and 7% styrene admixture. Qualitative identification of the microplastics used was obtained using a Nicolet iN10 FTIR microscope (ThermoFisher Scientific Inc., MA, USA) with a cooling detector for sample mapping. Infrared spectroscopy was performed in the reflectance mode. The collected spectra were analyzed using Omnic Spectra software with its database. Polypropylene and styrene granules with a diameter of 4.0 mm are used to prepare the substrate. Currently, nurseries are looking for alternatives to the previously used peat substrates. Alternative substrates are often prepared from organic remains from city parks containing microplastic particles. Fragments of crushed MCs are usually several mm in size, so we decided that a size of 4 mm would be the most appropriate in our study. In model experiments, various admixtures of MCs particles are used, usually from 1 to 20%. We decided that the use of two doses (a lower 2.5% and a higher 7%) would allow us to demonstrate the effect of the added types of MPs on the processes taking place in the medium in which the seedlings grow. Each variant of the experiment had three repetitions, including 53 seedlings. The experiment started on March 15, 2023 and lasted until September 30, 2023. No fertilisation was applied during the experiment, and irrigation was not differentiated. After the experiment, the seedlings were collected, cleaned and transported to the laboratory for further analysis. After the experiment, the substrate was collected to determine the enzymatic activity.

**Table 1:** Basic properties of the substrate used in the experiment.

pHH_2_O	pHKCl	N	C	C/N	Ca	K	Mg	Na	P	Al
3.67	2.58	0.87	46.65	53.62	4.01	1.39	1.68	0.03	387.2	2850.3

N and C content (%), base cations content (cmol( +)^.^kg^−1^), P and Al (mg kg^−1^).

### Laboratory analysis

The seedlings were divided into leaves, shoots and roots. In the leaves, shoots and roots samples, the concentration of macro- and microelements was determined using the abovementioned ICP spectrometer. Dried samples of leaves, shoots and roots were mineralised in a mixture of HNO_3_ and HClO_4_ (3:1). C and N content in the leaves, shoots and roots was measured with an elemental analyser (LECO CNS TrueMac Analyzer (Leco, St. Joseph, MI, USA)). The C/N, C/P and N/P ratios on a molecular level were calculated.

Substrate samples with natural moisture were taken to determine enzymatic activity. After being transported to the laboratory, the samples were stored in the fridge at 4 °C. Enzymatic activity was determined using the fluorescence method. The activity of six extracellular enzymes, namely β-glucosidase (BG), β-D-cellobiosidase (CB), β-xylosidase (XYL), N-acetyl-β-D-glucosaminidase (NAG), phosphatase (PH), and arylsulfatase (SP), was determined using the methodologies outlined by Pritsch et al^27^, Turner^28^, and Sanaullah et al.^29^. We recorded the fluorescence on a multidetection plate reader, with an excitation wavelength of 355 nm and emission at 460 nm.

The root samples, enclosed in a water cuvette, underwent analysis utilizing an Epson Expression 12000XL scanner (Epson America Inc., Long Beach, California) operating at a resolution of 800 dpi. The analysis was conducted through WinRHIZO Reg Software (version 2021, Regent Instruments Inc, Quebec, Canada) to ascertain their diameter, length, and root area. For the leaf samples, a flatbed scanner (Epson Perfection V800 Photo, Epson America Inc., Long Beach, California) was employed, scanning at a resolution of 600 dpi. Subsequent analysis was carried out using WinFolia Reg Software (version 2014, Regent Instruments Inc, Quebec, Canada) to determine the width, length, and leaf area. The air-dried roots were further desiccated at 70 °C for 24 h to a constant weight and then weighed. The root tissue density (RTD) (kg m^−3^), specific root area (SRA) (m^2 ^kg^−1^) and specific root length (SRL) (m g^−1^) were calculated according to Ostonen et al.^30^.

### Statistical analysis

The Shapiro–Wilk test was used to assess normality, and Levene’s test was used to check the homogeneity of variances. The Kruskal–Wallis test was used to assess the differences between the characteristics of the seedlings growing on different substrates. Two-way ANOVA was used to determine the role of sample type, microplastic type and amount and their interaction with the nutrient content in seedlings. Principal component analysis (PCA) was used to evaluate the relationships between the studied variables and group the tested substrate variants concerning enzymatic activity. Statistical analyses were performed using the programming language R (R Core Team, 2020) in R Studio (RStudio Team, 2020).

### Ethical approval

Our research on plants, including the collection of plant material, complied with relevant institutional and national legislation. We have the necessary permits to conduct research (Consent issued by the Forest District Manager in Wisła on March 13, 2023 (NG.5001.1.2023.AM). One-year-old beech seedlings were the voucher specimen. Prof Jarosław Lasota conducted the identification. Voucher specimens had not been deposited in a public collection due to the use of all material in laboratory analysis.

## Results

### Nutritional status of seedlings

The analyses indicate differences in the content of selected micro and macro elements (Table 2). The statistical analyses performed (2-way ANOVA) indicated significant differences in the content of the tested nutrients depending on the seedling's elements. The leaves were characterised by a significantly higher content of nutrients regardless of the type of substrate. The type of microplastic significantly influenced the content of nitrogen, calcium and potassium (Table 2). The quantity of microplastics in the substrate significantly influenced the aluminium content. The analyses also indicate a significant impact of the three studied variables (seeding element, MCs type and MCs amount) on the content of nitrogen, carbon, calcium and potassium (Table 2).

**Table 2:** The content of elements in different parts of seedlings growing on different substrates.

Seedling element	Variant	N	C	Al	Ca	K	Mg	P						
		g kg^−1^	mg kg^−1^											
Leaves	C	1.06 ± 0.07	46.00 ± 0.16	231.6 ± 30.6	6925.3 ± 158.4	3377.1 ± 426.1	1890.9 ± 86.6	386.3 ± 42.2						
	PP2.5	0.93 ± 0.03	45.62 ± 0.11	200.4 ± 12.4	6046.3 ± 80.7	2944.5 ± 313.8	1974.8 ± 78.5	376.1 ± 21.8						
	PP7	0.91 ± 0.07	45.59 ± 0.10	221.6 ± 45.6	6373.7 ± 262.6	2442.7 ± 425.9	2057.5 ± 167.5	364.6 ± 24.2						
	ST2.5	0.88 ± 0.05	45.76 ± 0.08	221.4 ± 15.1	6527.0 ± 384.6	2639.5 ± 222.2	2016.7 ± 155.3	381.2 ± 32.2						
	ST7	0.92 ± 0.05	45.71 ± 0.18	252.4 ± 58.5	6292.7 ± 217.9	2725.5 ± 20.9	2038.3 ± 72.0	388.4 ± 22.3						
Shoots	C	0.63 ± 0.06	45.22 ± 0.22	41.6 ± 7.6	4271.7 ± 248.2	2278.6 ± 118.8	1281.3 ± 103.0	374.4 ± 14.9						
	PP2.5	0.59 ± 0.04	44.57 ± 0.08	36.9 ± 8.8	4415.3 ± 248.1	2067.5 ± 22.3	1344.3 ± 53.1	389.0 ± 13.4						
	PP7	0.60 ± 0.05	44.38 ± 0.25	39.1 ± 6.8	4196.0 ± 227.3	2079.2 ± 16.5	1294.8 ± 53.0	397.9 ± 47.2						
	ST2.5	0.61 ± 0.06	45.01 ± 0.19	35.0 ± 5.1	4089.0 ± 419.4	1991.8 ± 102.3	1402.0 ± 116.4	410.2 ± 45.6						
	ST7	0.59 ± 0.02	44.82 ± 0.23	42.0 ± 12.4	4183.7 ± 197.2	2030.7 ± 73.9	1299.7 ± 33.5	407.7 ± 27.4						
Roots	C	0.41 ± 0.05	43.72 ± 0.51	14.9 ± 3.0	1592.9 ± 264.0	1947.2 ± 120.6	955.7 ± 79.8	247.7 ± 15.1						
	PP2.5	0.41 ± 0.04	43.84 ± 0.50	16.9 ± 3.3	1697.8 ± 95.6	1944.3 ± 56.3	939.5 ± 59.3	269.1 ± 7.9						
	PP7	0.45 ± 0.06	44.20 ± 0.76	20.4 ± 0.9	1863.8 ± 214.7	2121.0 ± 248.5	981.6 ± 0.8	293.9 ± 30.8						
	ST2.5	0.40 ± 0.02	44.07 ± 0.06	14.1 ± 2.4	1758.8 ± 170.3	2054.3 ± 189.3	909.6 ± 77.7	280.0 ± 8.6						
	ST7	0.42 ± 0.03	44.08 ± 0.47	16.1 ± 3.3	1804.5 ± 157.5	2039.0 ± 159.2	898.8 ± 73.4	289.2 ± 13.0						

2-way ANOVA	F	*p*	F	*p*	F	*p*	F	*p*	F	*p*	F	*p*	F	*p*
Seedling element (Se)	**261.02**	**0.0211**	**97.60**	**0.0000**	**248.21**	**0.0000**	**734.82**	**0.0000**	**70.59**	**0.0000**	**241.56**	**0.0000**	**66.99**	**0.0000**
MCs type (Mt)	**4.22**	**0.0000**	2.50	0.0924	1.86	0.1667	**4.20**	**0.0101**	**4.58**	**0.0465**	0.97	0.3855	1.24	0.2971
MCs amount (Ma)	0.38	0.5362	0.00	0.8637	**22.65**	**0.0106**	0.11	0.7415	0.18	0.6661	0.01	0.9279	0.46	0.4974
Se*Mt*Ma	**3.13**	**0.0236**	**4.73**	**0.0032**	0.93	0.4519	**3.04**	**0.0268**	**5.61**	**0.0001**	1.82	0.1421	1.67	0.1727

C–control, PP–polypropylene, ST–styrene; 7 and 2.5—amount of microplastics in %; significance effect *p* < 0.05.

**Figure 1:**
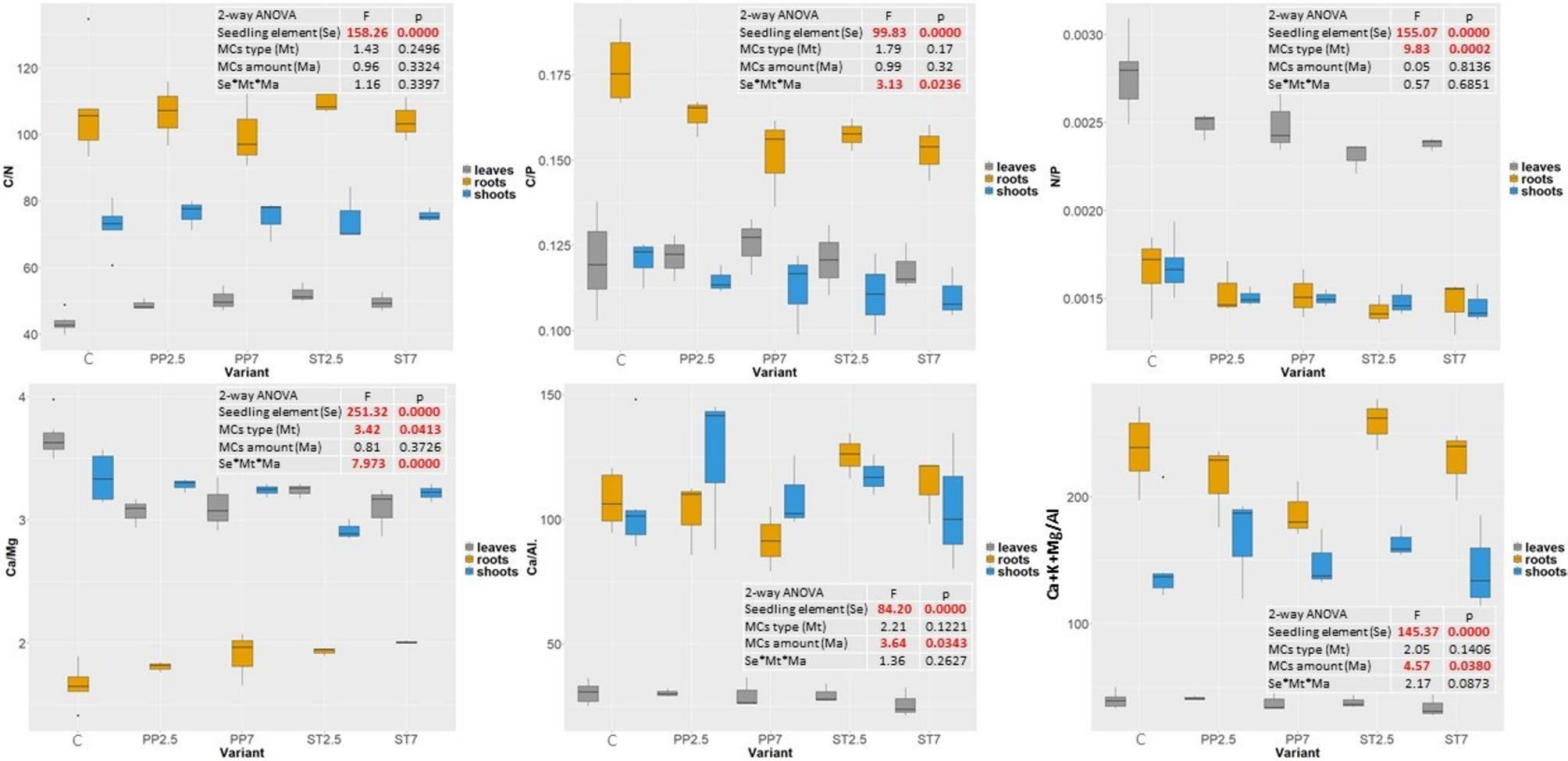
Molar ratios of elements in different parts of seedlings growing on different substrates (C–control, PP–polypropylene, ST–styrene; 7 and 2.5—amount of microplastics in %).

We noted significant differences in C/P and N/P depending on the seedlings element (Fig. 1). The type and amount of microplastics had no effect on the stoichiometry of C/N and C/P. In the case of the C/P ratio, the interaction of three factors, i.e., seedling element, MCs type, and amount, was important. The type of microplastic was important in shaping the N/P ratio. The highest N/P ratio was found in leaves. The highest N/P ratio was recorded in samples from the control variant, regardless of seedling element (Fig. 1). Seedlings element significantly influenced the C/Mg, Ca/Al and Ca^+^K^+^Mg/Al ratios. The Ca/Mg ratio varied depending on the type of microplastic, while the amount of microplastic was important in shaping the Ca/Al and Ca^+^K^+^Mg/Al ratios (Fig. 1).

### Root morphological features

**Figure 2:**
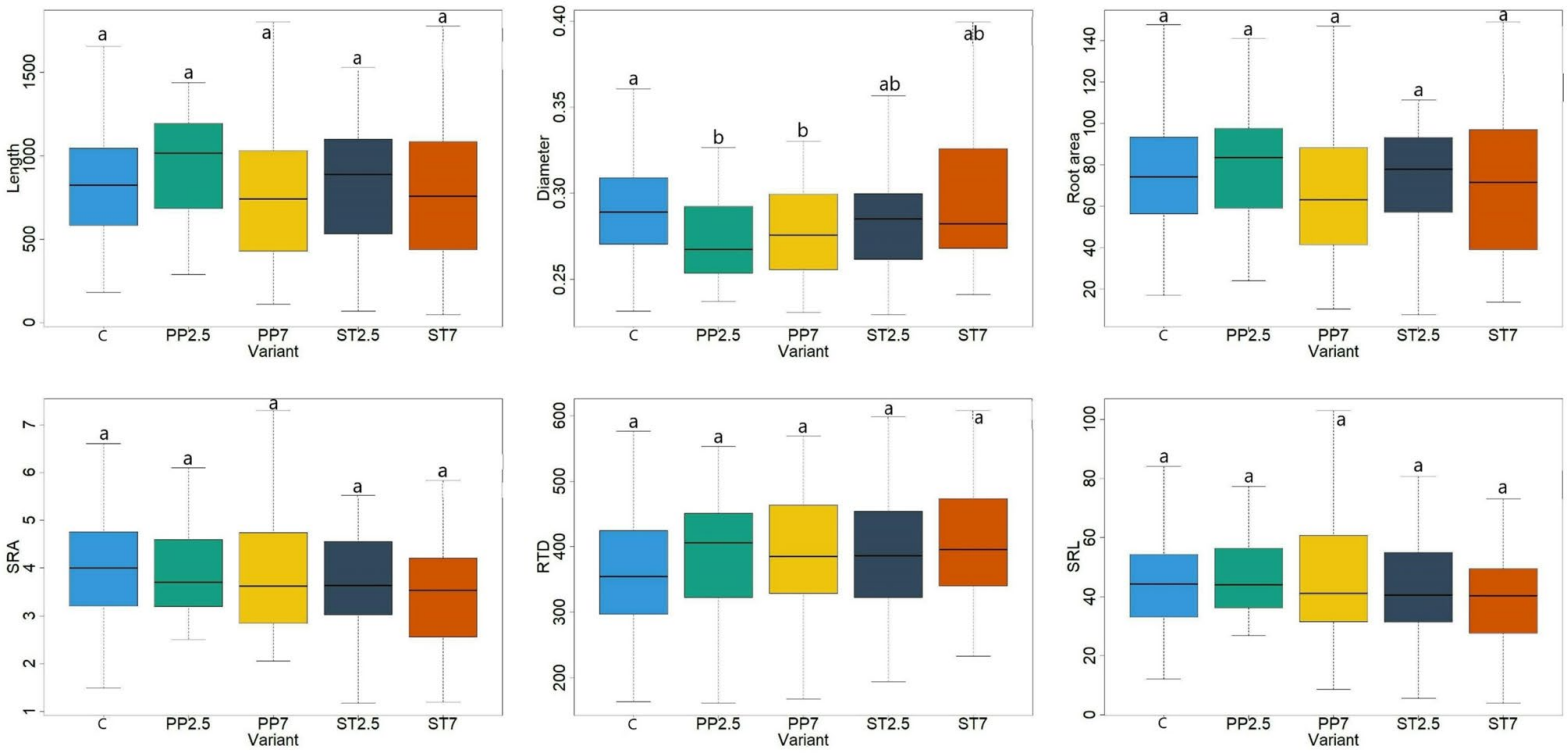
Morphological features of the roots of seedlings growing on different substrates (C–control, PP–polypropylene, ST–styrene; 7 and 2.5—amount of microplastics in %; length roots (cm), diameter roots (mm), root area (cm^2^), SRL—specific root area (m^2^ kg^−1^), RTD—root tissue density (kg m^−3^), SRA—specific root length (m kg^−1^), lowercase letters a and b indicate differences between the substrate).

**Figure 3:**
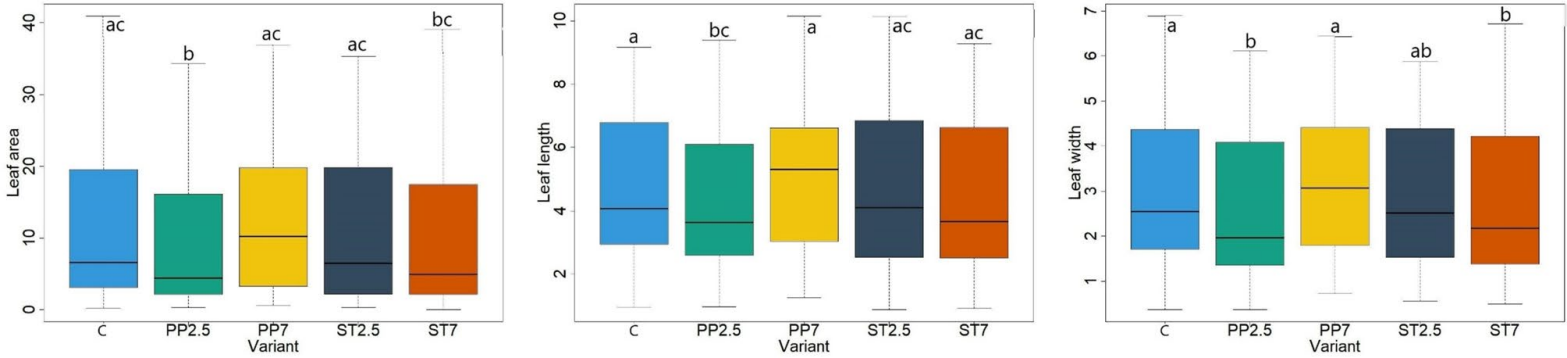
Detailed characteristics of the leaf of seedlings growing on different substrates (C–control, PP–polypropylene, ST–styrene; 7 and 2.5—the amount of microplastics in %; leaf area–cm^2^, leaf length and width–cm, lowercase letters a and b indicate differences between the substrate).

The analysis of the root systems of beech seedlings showed smaller differences depending on the substrate on which they grew (Fig. 2). There were no statistically significant differences in most of the examined root characteristics, i.e. length, root area, SRA, RTD and SRL. Significantly thicker roots of seedlings in control plots were recorded compared to variants with polypropylene (Fig. 2). After analysing the leaves of beech seedlings, differences in their characteristics were found depending on the substrate on which they grew. The leaf area was significantly larger in the control variant compared to the variant with polypropylene in the amount of 2.5% and styrene in the amount of 7%. The length and width of leaves were significantly higher in the control variant compared to the PP2.5 and ST7 variants (Fig. 3).

### Enzymatic activity of the substrate

**Figure 4:**
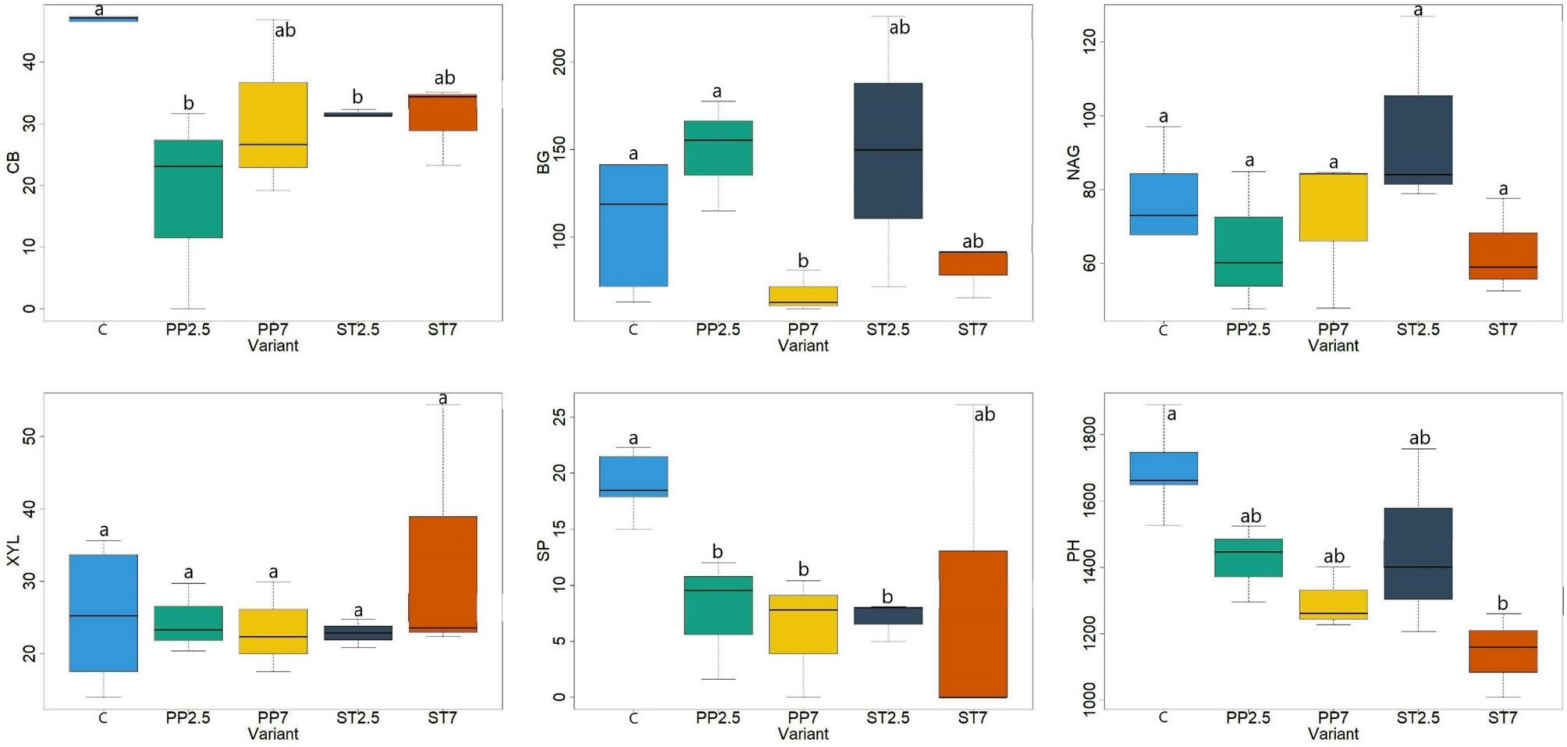
Enzymatic activity (nmol MUB g^−1^ ·h^−1^) CB–β-D-cellobiosidase, BG–β-Glucosidase, NAG–N-acetyl-β-Glucosaminidase, XYL–β-Xylosidase, SP–Sulphatase, PH–Phosphatase (C–control, PP–polypropylene, ST–styrene; 7 and 2.5—amount of microplastics in %; lowercase letters a and b indicate differences between the substrate).

**Figure 5:**
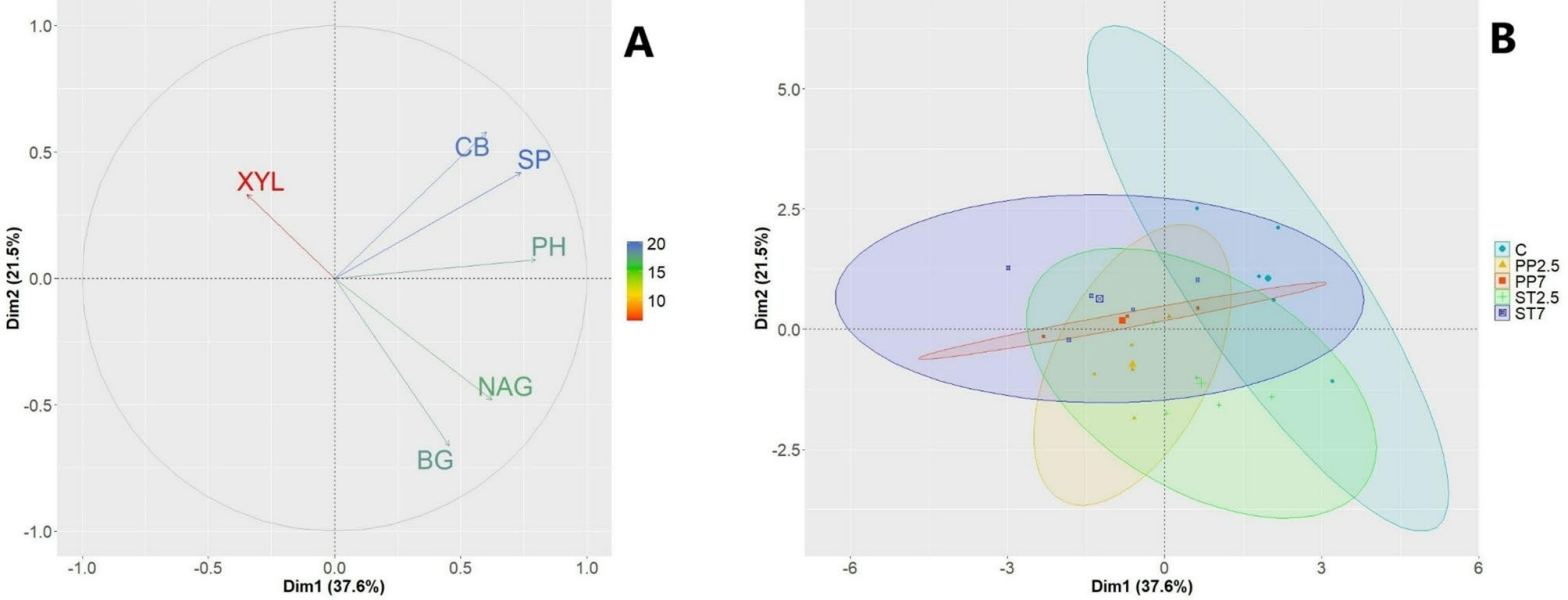
The projection of variables on a plane of the first and second PCA factors in the seedlings growing on different substrates (C–control, PP–polypropylene, ST–styrene; 7 and 2.5—amount of microplastics in %).

The analysis of the enzymatic activity of the substrate on which the seedlings grew indicates differences depending on the variant (Fig. 4). The control variant had the highest CB, SP and PH activity compared to the other variants. In the case of CB and SP, the activity was twice as high in the control variant compared to the variants with microplastics. In the case of BG, the activity in the control variant was higher in relation to the PP7 and ST7 variants. In the case of NAG, activity did not differ significantly between the variants included in the analysis (Fig. 4). Factors 1 and 2 of the PCA analysis explain approximately 60% of the variability of the presented features. At the same time, PCA analysis confirms the distinctiveness of the controllable substrate in terms of enzymatic activity compared to other substrate variants used in the experiment (Fig. 5).

## Discussion

### Nutritional status and root morphological features

The results of our experiment indicate that both the type of added microplastics (polypropylene and styrene) and their amount are important for the development of seedlings during one growing season. Other researchers have reached similar conclusions^31^. In our experiment, the type of microplastic that had a more visible effect on beech growth was PP. In the case of the addition of this type of microplastics, we observed a more significant deterioration in indicators reflecting the nutritional status of cultivated beech seedlings. This was especially visible in relation to the proportion of basic cations to aluminium (Ca^+^K^+^Mg: Al and Ca: Al) in beech leaves. The higher admixture of PP in the substrate resulted in a particularly visible deterioration of these indicators. The indicators analysed both in soil solutions and plant material are good measures of growth conditions as well as the nutritional status of plants^32–34^. The literature indicates certain thresholds that may be alarming and beyond which (reduction of the value < 1) symptoms of disturbances in physiological processes may occur. What could be the reason for such significant changes in plant nutrition related to the presence of PP in the substrate? We assume that stress caused in plants by microplastic particles has two main causes—blocking the pores during seed germination and in the roots, hindering the uptake of water and nutrients, and causing drought as a result of the intensification of the substrate cracking process in the presence of microplastic particles which was indicated in previous studies^18,22,35^. In the case of our experiment, ionic balance disturbances in plants should probably be associated with the negative impact of microplastics on the uptake of water and mineral salts. In our experiment, we used microplastics with dimensions of 4 mm, which may impede the flow of water and with it nutrients. The second reason should rather be rejected. Krehl et al^35^ proved that microplastics have a significant impact on the soil structure and increase drought stress during periods of water shortage. In the above-mentioned experiment, the impact of microplastics on the water content and plant response depended on the soil texture. In the case of clay soils, a small addition of MCs (0.1%) resulted in water content, which the authors explained by improving the structure and, at the same time, water availability. At higher MCs contents (0.5 and 1%), there was a negative effect on water availability, which indicates a deterioration of the soil structure and the formation of pores where water percolation or evaporation occurs. In the case of sandy soils, the addition of MPs in any amount resulted in a deterioration of water availability for growing plants. In our case, an organic substrate with high porosity, as a result of adding MCs in the amount of 2.5 and 7%, certainly increased the number of macropores, which probably disturbed the flow of water and nutrients. In our experiment, the seedlings were regularly watered to prevent them from experiencing drought stress. It is difficult to clearly indicate the reasons for the more negative impact of polypropylene particles on cultivated beech seedlings compared to styrene. There are reports that styrene molecules can be more easily taken up by the roots of herbaceous plants, e.g. in relation to polymethyl methacrylate. Contact with styrene microparticles induces the activation of antioxidant mechanisms in root cells (increase in the activity of catalase and peroxidases), which protects against (cyto)toxic effects on root growth^36^. In our experiment, we observed no visible effect of the added types of microplastics on the characteristics of beech root systems. An interesting phenomenon is the decrease in the C/P and N/P ratios in the stems and roots of beech trees growing on the substrate with the addition of both types of microplastics. This is due to the tendency to increase the P content in the stems and roots of seedlings grown in the substrate with the addition of microplastics, which would indicate an increase in the uptake of this macronutrient under the experimental conditions. In acidic soils and substrates, the availability of phosphorus and its absorption is closely related to the metabolic activity of mycorrhizal fungi living in symbiosis with the roots. There are reports that the addition of microplastics such as (PA, PEHD, PET, PP and PS) did not increase colonisation by mycorrhizal fungi^31^. At the same time, there is evidence that the addition of microplastics may change the population structure of soil bacteria towards the growth of taxa that have enzymatic abilities that favour the mineralisation of soil P^37–39^.

### Enzymatic activity of the substrate

During a several-month experiment, we assessed the impact of various types and amounts of microplastics on the enzymatic activity of the substrate on which beech seedlings grew. Soil enzymes produced by microorganisms have a significant role in the soil, actively participating in the biogeochemical cycles of carbon (e.g. β-glucosidase), nitrogen (e.g. urease) and phosphorus (e.g. phosphatase)^40^. They are considered to be sensitive to various environmental stressors, which makes them important bioindicators assessing soil health^41^. Previous research has shown that the introduction of microplastics can increase, decrease, or have no effect on enzyme activity in soil; the diversity of these effects may be related to the type, amount and duration of exposure to microplastics, as well as the characteristics of the soil itself^42^. According to the authors cited above, the addition of polypropylene (PP) increased the enzyme activity by 10.2%, respectively, while exposure to polyethylene terephthalate (PET), polyethylene (PE) and polystyrene (PS) decreased the soil enzyme activity by 13.0%, respectively 6, 8% and 5.0%, while soil enzyme activity was stimulated and inhibited when MPs concentrations were lower and > 10% and MPs exposure not significantly (p > 0.05) affect soil enzyme activity, respectively. Our analyses indicate the inhibition of the activity of selected enzymes as a result of the impact of both polypropylene and styrene microplastics. Significantly lower activity in the microplastic variants was recorded in the case of CB, SP and PH and to a lesser extent in the case of BG. The presence of microplastics can affect the contents and forms of soil carbon and nitrogen nutrients and the emissions of CH_4_, CO_2_, and N_2_O by altering soil microbial communities, functional gene expressions, and enzyme activities^43^. Research by Lian et al.^44^ indicates that microplastics reduced phytase activity and significantly increased dehydrogenase activity, and at the same time, the diversity and structure of the rhizosphere microbial community were disrupted. Additionally, microplastics may affect the stability of the bacterial coexistence network in the soil and disrupt the metabolic processes of microorganisms, such as nitrogen fixation and urea degradation^45^. Important processes related to the nitrogen cycle are directed by microorganisms, with a key role played by enzymatic reactions carried out by a number of important enzymes that are encoded by appropriate functional genes^46^. Based on the results obtained, it can be concluded that research on the impact of microplastics on soil properties and the growth of tree seedlings should be continued. Future research should take into account the impact of microplastics depending on soil type and tree species.

Our study substantiated the crucial role of microplastics in shaping the nutritional status of young beech trees, evident after just one vegetation period. There was a notable influence of the microplastic variety on the N/P and Ca/Mg ratios, and the quantity of microplastic affected the Ca/Al and combined Ca^+^K^+^Mg/Al ratios. In terms of root diameter, the control group exhibited significantly thicker roots, while microplastics were influential in defining the leaf attributes of the examined species. The leaf area was significantly larger in the control variant compared to the variant with polypropylene in the amount of 2.5% and styrene in the amount of 7%. Furthermore, our research highlights the substantial effect of microplastics on enzyme activity. This research adds valuable data to the global pool of knowledge regarding microplastics. The results of our research may help in the development of new guidelines for planting, cultivation, and protection of forests in polluted areas. Highlighting the effects of microplastics on something as tangible as tree growth can raise public awareness about environmental pollution. This, in turn, can drive policy changes and encourage more sustainable plastic use and waste management practices.

## Data Availability

The datasets generated and analysed during the current study are not publicly available due to legal reasons but are available from the author upon reasonable request. Contact person – Ewa Błońska (ewa.blonska@urk.edu.pl).
